# Highlights from ASCO-GI 2021 from EORTC Gastrointestinal tract cancer group

**DOI:** 10.1038/s41416-021-01474-y

**Published:** 2021-08-23

**Authors:** Thibaud Koessler, Maria Alsina, Dirk Arnold, Irit Ben-Aharon, Manfred P. Lutz, Radka Obermannova, Mark Peeters, Francesco Sclafani, Elizabeth Smyth, Juan W. Valle, Anna Dorothea Wagner, Lucjan Wyrwicz, Elisa Fontana, Markus Moehler

**Affiliations:** 1 Department of Oncology, Geneva University Hospital, Geneva, Switzerland; 2 Swiss Cancer Center Leman (SCCL), University of Geneva, Lausanne, Switzerland; 3 European Organisation for Research and Treatment of Cancer, Brussel, Belgium; 4 Vall d’Hebron University Hospital, Department of Medical Oncology, and Vall d’Hebron Institute of Oncology (VHIO), Universitat Autònoma de Barcelona, Passeig de la Vall d’Hebron, Barcelona, Spain; 5 Department of Oncology, Haematology and Palliative Care, Asklepios Klinik Altona, Asklepios Tumorzentrum Hamburg, Hamburg, Germany; 6 Division of Oncology, Rambam Health Care Campus, Rappaport Faculty of Medicine, Technion, Haifa, Israel; 7 Caritasklinikum, Saarbrucken, Germany; 8 Department of Comprehensive Cancer Care, Masaryk Memorial Cancer Institute and Faculty of Medicine, Masaryk University, Brno, Czech Republic; 9 Department of Pharmacology, Faculty of Medicine, Masaryk University, Brno, Czech Republic; 10 Department of Oncology, Universitair Ziekenhuis Antwerpen, Antwerp, Belgium; 11 Department of Medical Oncology, Institut Jules Bordet—Université Libre de Bruxelles (ULB), Brussels, Belgium; 12 Cambridge University Hospitals NHS Foundation Trust, Addenbrooke’s Hospital, Cambridge, UK; 13 Division of Cancer Sciences, University of Manchester, Manchester, UK; 14 Department of Medical Oncology, The Christie NHS Foundation Trust, Manchester, UK; 15 Department of Oncology, Division of medical Oncology, Lausanne University Hospital (CHUV) and University of Lausanne (UNIL), Lausanne, Switzerland; 16 Maria Sklodowska-Curie Memorial Cancer Center and Institute of Oncology, Warsaw, Poland; 17 Sarah Cannon Research Institute, London, UK; 18 Department of Internal Medicine, Johannes-Gutenberg University, Mainz, Germany

**Keywords:** Biliary tract cancer, Gastric cancer, Colorectal cancer, Cancer immunotherapy, Drug development

## Abstract

Last year the field of immunotherapy was finally introduced to GI oncology, with several changes in clinical practice such as advanced hepatocellular carcinoma or metastatic colorectal MSI-H. At the virtual ASCO-GI symposium 2021, several large trial results have been reported, some leading to a change of practice. Furthermore, during ASCO-GI 2021, results from early phase trials have been presented, some with potential important implications for future treatments. We provide here an overview of these important results and their integration into routine clinical practice.

## Highlights in oesophagogastric cancer

### EORTC oesophagogastric task force: Elizabeth Smyth and Dorothea Wagner

**Fig. 1:**
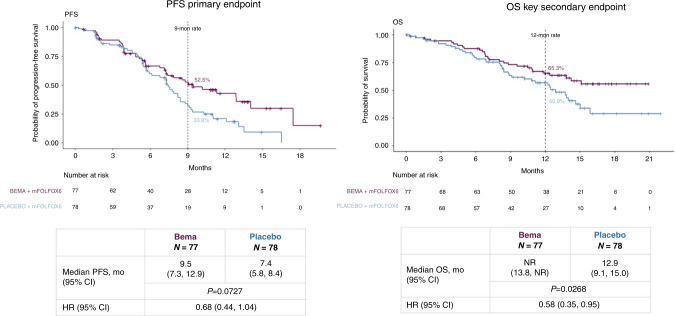
Progression-free survival and overall survival from the FIGHT trial: bemarituzumab plus mFOLFOX6 versus placebo plus mFOLFOX6. Abstract 160 presented by Dr Z Wainberg at 2021 ASCO Gastrointestinal Cancers Symposium [3]. Reproduction with the permission of the author.

ASCO-GI 2021 provided some interesting results in the areas of targeted therapy, and immunotherapy for patients with advanced gastric cancer. The first pathway addressed was the fibroblast growth factor receptor (FGFR) pathway, a potential therapeutic target which is known to stimulate angiogenesis, transformation and proliferation of tumour cells [1]. Bemarituzumab is a first-in class humanised IgG1 monoclonal antibody, which selectively binds to FGFR2b. On the basis of a favourable safety profile and an 18% confirmed overall response rate (RR) in a phase I-study in pre-treated patients with gastroesophageal cancer, FIGHT (NCT03343301), a global, double-blind, randomised phase II trial evaluating Bemarituzumab was conducted in chemo naive patients with HER2 negative, advanced or metastatic GC and centrally confirmed either FGFR2b overexpression by immunohistochemistry or -amplification by circulating tumour DNA [2]. The design of this trial was changed from phase III to a randomised phase II while ongoing. One hundred and fifty-five patients were randomised 1:1 to mFOLFOX6 with either bemarituzumab 15 mg/kg or placebo every 2 weeks plus 7.5 mg/kg bemarituzumab or placebo on day 8. The primary endpoint was progression-free survival (PFS), among the secondary endpoints were overall survival (OS) and ORR. Of 910 patients tested, 30% were FGFR2B positive. With a median PFS of 9.5 vs. 7.4 months (hazard ratio [HR] 0.68, 95% confidence interval [CI], 0.44–1.04, *p* = 0.07) Fig. 1, and a median OS not reached (NR) in patients treated with bemarituzumab vs. 12.9 months (HR 0.58, 95% CI, 0.35–0.95, *p* = 0.03), both endpoints were met. Stomatitis and corneal adverse events (AEs) were higher in patients treated with bemarituzumab. While this proof-of-concept study clearly demonstrates activity of FGFR2b targeting in gastric cancer, longer follow-up and confirmation of these findings in a phase III trial are necessary to fully characterise the magnitude of the patient benefit from this treatment [3].

HER2-directed therapy was also in the spotlight at GI ASCO 2021 with promising signs of efficacy for a novel HER2-targeted bispecific antibody zanidatamab in gastric cancer. Zanidatamab monotherapy showed a RR of 38%, and was also safely combined with chemotherapy [4]. Together with trastuzumab deruxtecan and margetuximab, zanidatamab appears to be a good candidate for integration with chemotherapy or immunotherapy in future for HER2 positive gastric cancer patients [5, 6]. Considering HER2-directed therapy plus immunotherapy, the results of triplet treatment of chemotherapy, trastuzumab and pembrolizumab in first-line advanced gastric cancer in the PANTERA study were also presented at ASCO-GI 2021 [7]. Radiological response rates of 76% were observed, with encouraging median OS of 19.3 months (95% CI, 16.5-NR). These results are consistent with those observed in a single centre US study [8]. This approach is currently under investigation in the global KEYNOTE-811 trial. In contrast, the results of the global LEAP-005 study of lenvatinib and pembrolizumab in previously treated gastric cancer presented at ASCO-GI 2021 were markedly different from those presented previously in a single centre Japanese study [9, 10]. In the latter, radiological RR of 69% (95% CI, 49–85) were reported, whereas in the global LEAP-005 trial the objective RR was <10%, highlighting the importance of international trials evaluating the efficacy of new drugs for gastric cancer patients. Finally, updated results of the JACCRO-07 study were presented. These confirmed not just a recurrence-free survival benefit but also an overall survival benefit for the addition of docetaxel to adjuvant S1 chemotherapy [11]. This validates docetaxel-S1 as a therapeutic option for resected gastric cancer in Asian patients.

Overall, although the findings at GI ASCO were not practice changing for patients with advanced oesophagogastric cancer, they do offer hope for the development of effective therapies in future. Whether targeted therapies such as anti-HER2 or anti-FGFR2b will be combined with immune checkpoint blockade is the emergent critical question.

## Highlights in hepatocellular carcinoma, biliary tract cancer, neuroendocrine tumours and pancreatic cancer

### EORTC hepatobiliary, pancreas and NET task force: Juan W. Valle, Manfred P. Lutz and Jens Ricke

#### Cholangiocarcinoma (CCA)

Dr Zhu presented the final OS results of the randomised phase III ClarIDHy study wherein 187 patients with previously treated isocitrate dehydrogenase-1-mutated (mIDH1) CCA received either ivosidenib (an oral inhibitor of mIDH1, *n* = 126) or placebo (*n* = 61) [12]. The study had previously met its primary endpoint, PFS: HR 0.37, 95% CI, 0.25–0.54, one-sided *p* < 0.0001] [13]. OS, a secondary endpoint, was confounded by protocol-enabled cross-over to ivosidenib of placebo-treated patients on disease progression, occurring with 43/61 (70.5%) of patients. The final unadjusted median OS (mOS) was 10.3 vs. 7.5 months, HR 0.79, 95% CI, 0.56–1.12, one-sided *p* = 0.093; however after pre-specified adjustment for cross-over (using the rank-preserving structural failure time model), the mOS was 10.3 vs. 5.1 months, HR 0.49, 95% CI, 0.34–0.70, one-sided *p* < 0.0001. The treatment had a tolerable safety profile and patients receiving ivosidenib had preserved physical functioning and improved pain scores on QoL measures. IDH1 mutation are found in 13% of intrahepatic cholangiocarcinoma.

Dr Javle presented the final results of the phase II study of oral infigratinib (BGJ398) in patients with previously treated CCA harboring fibroblast growth factor receptor-2 (FGFR2) fusions and rearrangements. These activating translocation events are found in 20% of intrahepatic cholangiocarcinomas. This was deemed to be an active regimen with a centrally confirmed RR of 23.1% (95% CI, 15.6–32.2), median duration of response 5.0 months (95% CI, 0.9–19.1), PFS 7.3 months (95% CI, 5.6–7.6) and OS 12.2 months (95% CI, 10.7–14.9); subgroup analysis suggested that less heavily pre-treated patients (≤1 lines of treatment) derived greater benefit. Although the commonest fusion partner was BBICC1, 35% of patients had a novel fusion partner. Adverse events (mostly mechanism-based) were predominantly grade 1–2 and manageable [14].

In her discussion, Dr Shroff highlighted the importance of clinical benefit measures (e.g. impact on QoL) to patients in addition to efficacy endpoints; and the emerging questions around availability, optimal timing, selection (in the setting of multiple agents), potential combinations, sequencing and identification of resistance mechanisms of targeted therapies in cholangiocarcinoma, which she described as “the poster child for precision medicine”.

Our understanding of cholangiocarcinoma pathogenesis makes it, currently, the digestive cancer with the most numerous druggable driver alterations. These mutations, amplifications, and fusions should be systematically looked for as treatment options are now available. Indeed, advanced cholangiocarcinoma treatment landscapes evolve quickly with several new compounds—almost all being tyrosine kinase inhibitors (TKI). As with other TKIs, their effect is short-lived with rapid development of resistant mutations. Treatment landscape is moving towards increasing the duration of effectiveness of these compounds—by combining TKIs with immune checkpoint inhibitors or with chemotherapy—as well as developing a second generation of TKIs.

#### Hepatocellular carcinoma

##### IMBrave150 study survival update

**Fig. 2:**
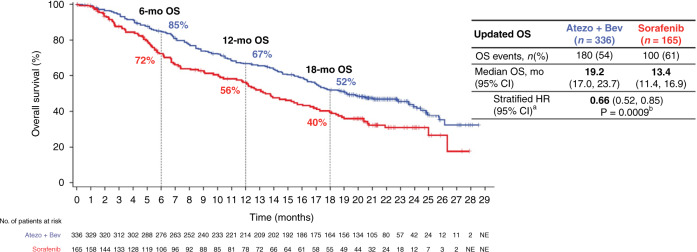
Updated overall survival data from the IMbrave150 study: atezolizumab + bevacizumab versus sorafenib in patients with unresectable hepatocellular carcinoma. Abstract 267 presented by Dr R Finn at 2021 ASCO Gastrointestinal Cancers Symposium [16]. Reproduction with the permission of the author.

The results of this first-line study comparing atezolizumab and bevacizumab (atezo-bev) vs. sorafenib have been published leading to the approval atezo-bev for first-line treatment of advanced HCC [15]. At the time of the publication, the mOS was “NR” vs. 13.2 months, HR 0.58, 95% CI, 0.42–0.79, *p* < 0.001, in favour of atezo-bev. With an additional 12 months of follow-up (median duration of follow-up 15.6 months) the mOS is 19.2 vs. 13.4 months, HR 0.66, 95% CI, 0.52–0.85, *p* = 0.0009, with early and sustained separation of the survival curves, Fig. 2. Updated PFS is 6.9 vs. 4.3 months HR 0.65, 95% CI, 0.53–0.81, *p* = 0.0001; the response rate in atezo-bev-treated patients was 30% including 8% complete responses (RECIST) [16].

##### KEYNOTE-240 update

With an additional 18 months of follow-up of the previously-reported study (median 40 months follow-up), the updated OS was numerically maintained (and very similar) in patients receiving pembrolizumab (vs. placebo, in second-line for sorafenib-treated patients with advanced HCC): 13.9 vs. 10.6 months (HR 0.77, 95% CI, 0.62–0.96, *p* = 0.0112) [17]. The PFS was also maintained: 3.3 vs. 2.8 months (HR 0.70, 95% CI, 0.56–0.89, *p* = 0.0011) with a superior RR (18.3% vs. 4.4% with placebo) [18].

##### CheckMate-040 update

Four cycles of 3-weekly nivolumab 1 mg/kg and ipilimumab 3 mg/kg followed by 2-weekly nivolumab 240 mg was approved in the USA for patients with disease progression after sorafenib based on a RR of 32% (95% CI, 20–47%, RECIST v1.1) and OS 22.8 months (95% CI, 9.4–NR) [19]. With long-term follow-up (median 46.5 months), this activity is maintained with RR 32% (95% CI, 20–47%, unchanged) with duration of response 17.5 months (95% CI, 8.3–NR); OS 22.2 months (95% CI, 9.4–NR); and 3-year survival 42%. No new safety signals were seen [20].

##### TACTICS study (TACE +  sorafenib vs. TACE alone) update

A co-primary endpoint for this study (PFS) has previously been published: 25.2 vs. 13.5 months in favour of the combination (HR 0.59, *p* = 0.006) [21]. Results of OS, the other co-primary endpoint, were presented. With a median follow-up of 33.4 months, the median OS was 36.2 (combination) vs. 30.8 months (TACE alone); HR 0.86, 95% CI, 0.61–1.22, *p* = 0.40. A greater proportion of patients randomised to TACE alone received subsequent therapies (76.3% compared to 58.8%) which potentially confounded the OS analysis as the PFS remained statistically significantly in favour of the combination. The authors argued that OS is no longer an appropriate endpoint for TACE-based studies [22].

In conclusion, immune checkpoint inhibition is now part of the advanced hepatocellular carcinoma treatment landscape, its role in earlier settings such as post resection or in association with local treatments is heavily studied. In advanced disease, the importance of the sequence now becomes critical, bearing in mind that 40% of patients will not be able to have a second line of treatment, and in light of the recent analysis published by Pfister et al. showing that hepatocellular carcinoma of non-alcoholic steatohepatitis origin might be less responsive to immunotherapy [23].

#### Neuroendocrine tumours (NET)

The results of the phase II/III AXINET study were presented by Dr Garcia-Carbonero. Patients (*n* = 256) with pre-treated (≤2 lines of prior therapy), progressive, well-differentiated grade (G)1–2 extra-pancreatic NETs were randomised (1:1, stratified by time from diagnosis, gastrointestinal vs. non-gastrointestinal and by Ki67 ≤ 5% vs. >5%) to receive octreotide LAR with either oral axitinib (a potent VEGFR1–3 inhibitor) or placebo. The primary endpoint was not met: median investigator-assessed PFS 17.2 vs. 12.3 months (HR 0.82, 95% CI, 0.61–1.09, *p* = 0.0169), although patients receiving axitinib has a superior radiological response rate (17.5% vs. 3.8%, *p* = 0.0004). Blinded independent central review is ongoing. The safety profile was as expected from axitinib in other indications. Exploratory subgroup analysis suggests that patients with G1 NETs may derive greater benefit [24]. The discussant (Dr Snyder) advised caution in interpreting the G1 subgroup due to the small size of the subgroup (*n* = 22). She also set the results in context with other available therapies, primarily peptide receptor radionuclide therapy and everolimus, and highlighted that the role of multi-targeted tyrosine kinase inhibitors in these patients remains an active area of investigation.

Peptide receptor radionuclide therapy (PRRT) with Lu 177-dotatate is standard of care for well-differentiated (WD) NETs following progression on somatostatin analogues. Das et al, build a clinical score predicting outcome for patients with WD NETs receiving PPRT. The clinical score includes 5 variables: available non-PRRT treatments for tumour type, prior systemic treatments, patient symptoms, tumour burden in critical organs and peritoneal carcinomatosis presence each variable being score from 0 to 2 except peritoneal carcinomatosis from 0 to 1. For each 2-point increase in clinical score, the estimated HR for PFS is 3.26 (95% CI, 2.05–5.19) [25]. Coffman et al shared their experience on PRRT in WD high grade (HG), average tumour Ki67: 34.8% NETs with 63% partial response, 6% stable disease and 31% progressive disease amongst 19 treated patients [26].

Al-Toubah et al. treated 34 patients with HG neuroendocrine neoplasms with dual checkpoint inhibitor therapy—anti-PD-1 and anti-CTLA-4. Most patients (79.4%) had poorly differentiated NECs and 20.6% had WD HG NETs. With ORR: 14.7%, DCR: 41.2% and median PFS: 1 month, dual checkpoint inhibitor therapy has modest activity in HG, pre-treated neuroendocrine neoplasms [27]. In the PLANET, phase Ib/II trial, pembrolizumab and lanreotide were administered at 22 patients having progressed on somatostatin analogues. No response was observed, 40% had partial response and 50% progressive disease. Median PFS was 5.4 months and median OS not reached at 15 months of follow-up [28].

To conclude in the field of WD NETs, PRRT is a corner stone, and its effectiveness can now be predicted with clinical score. PRRT is currently being combined with chemotherapy or immunotherapy in different trials. Results on PRRT using somatostatin antagonist either for diagnostic or for therapeutics (e.g. ^177^Lu-OPS201) is eagerly awaited. Currently immunotherapy plays no role; however, it might change judging by the 40% partial response in the PLANET trial.

#### Pancreatic cancer

Neoadjuvant treatment of borderline resectable pancreatic adenocarcinoma is increasingly used worldwide and has even become part of the recommended standard treatment approach in the United States (NCCN 2020). The Alliance A021501 phase II trial was designed to select a reference regimen for future trials. Chemotherapy with mFOLFIRINOX (8 cycles) with or without radiotherapy (mostly SBRT, up to 40 Gy in 5 fractions) before surgery and adjuvant FOLFOX 6 (4 cycles) were evaluated in comparison to historical controls. The SBRT arm had to be closed after a planned interim analysis because of a poor R0 resection rate of only 25%. In the mFOLFIRINOX arm (*n* = 70), the R0 resection rate reached 42%, with a median OS of 31 months. The 18 months survival rate of 66.4% compares favourably with the pre-specified rate in historical controls of 50%, reinforcing mFOLFIRINOX as the reference for future trials [29].

In advanced disease, the PARP inhibitor olaparib has already been introduced as optional maintenance treatment by the POLO phase III trial, at least for patients with germline BRCA mutations and after disease stabilisation under platinum-based therapy [30]. The updated final overall survival analysis confirmed that OS was similar in the olaparib and placebo arm (19.0 vs. 19.2 months), with HR 0.83, 95% CI, 0,56–1,22, *p* = 0.349 [31]. The main benefit of olaparib was a strong trend in PFS2 (16.9 vs. 9.3 months, HR 0.66, 95% CI 0,43–1.02, *p* = 0.061), which is driven by long-term effects in the olaparib arm (rate of survival without progression after 36 months of 31.2% with olaparib vs. 13.1% for placebo). These results seem to be independent from the type of BRCA mutation and are also valid when the primary tumour—and not the metastases—are used as target lesion [32, 33].

Several groups presented feasibility data for cfDNA measurements. Tumour mutation burden (TMB) could be analyzed in 173/174 (99.4%) plasma samples of the PA.7 trial, which examined the efficacy of combined PD-L1 and CTLA-4 inhibition with gemcitabine and Nab-paclitaxel [34]. Only one patient was MSI-H with a TMB of 52.9 muts/Mb. Cut-point analysis defined ≥ 9 muts/Mb (4.6% of the patients) as predictive for increased OS with combined checkpoint inhibition. In another retrospective analysis, comprehensive genomic profiling of cfDNA detected at least one somatic alteration in 613/1009 (60.8%) of the examined samples [35]. Most common were gene alterations in *TP53* (53.7%), *KRAS* (40.3%), *CDKN2a* (6.5%), and in genes of the homologous recombinant DNA damage repair pathway (HR-DDR) in 12.3%. In addition, serial measurements with clearance of mTP53 or mKRAS cfDNA seemed to be predictive of increased PFS (HR 0.087 and 0.32 with *p* = 0.0056 and *p* = 0.037, respectively) in a small retrospective analysis of 23 patients under systemic treatment [36].

To conclude, in locally advanced and borderline pancreatic cancer, neoadjuvant chemotherapy is now clearly recommended. Chemotherapy choice encompasses FOLFIRINOX, mFOLFIRINOX or gemcitabine and albumin-bound paclitaxel. The benefit of PARP inhibitor, Olaparib, is confirmed in PFS in patients with germline BRCA mutations; however, the lack of survival and quality of life benefit should push clinicians to weigh the risk/benefit carefully.

## Highlights in anal and localised colorectal cancer

### EORTC anal and localised colorectal cancer task force: Dirk Arnold and Lucjan Wyrwicz

**Fig. 3:**
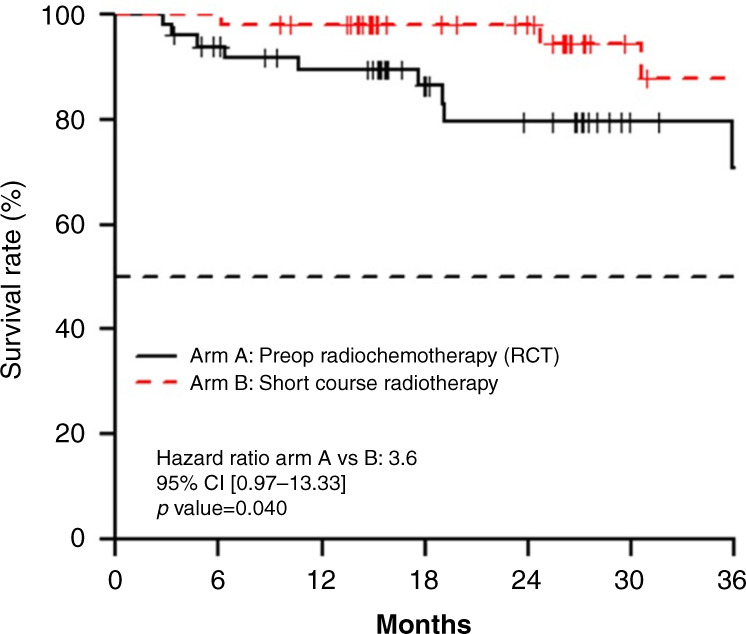
Overall survival from the NACRE trial: short course radiotherapy versus radiochemotherapy for locally advanced rectal cancers in the elderly. Abstract 4 presented by Dr E François at 2021 ASCO Gastrointestinal Cancers Symposium [37]. Reproduction with the permission of the author.

Previously, two independent studies showed the equal efficacy of short course radiotherapy and chemoradiation in locally advanced rectal cancer. There is the issue of excessive mortality in treatment of frail and elderly patients who undergo total mesorectal excision (TME). NACRE study presented by Eric Francois et al. focused only on elderly patients (>74 years old) randomising them to either short course RT or chemoradiation. Oncological outcomes were similar in both groups, with well-defined trends in 6- and 12-months excessive deaths in the chemoradiation arm (10% vs 3.9% at 6 months: 12% vs 3.9% at 12 months), Fig. 3. This observation should be verified in other datasets but can be applied especially in the case of frail elderly patients [37].

An important clinical question is whether patients with less advanced rectal cancer can undergo treatment and have a substantial chance of true organ preservation (i.e. watch-and-wait approach or local excision without TME). The preliminary outcomes of OPERA study presented by Sun Myint A. et al. has shown an organ preservation rate of more than 80% in cT2/T3 N0/1 patients treated with either chemoradiation (45 Gy) with additional external beam boost (9 Gy) or contact X-ray brachytherapy (90 Gy). Blinded combined outcomes of both arms were presented with only 19.4% patients who needed TME surgery throughout the observation period [38].

The major trend observed in rectal cancer is associated with a broad implementation of total neoadjuvant therapy (TNT) into clinical practice. This was reflected in the number of abstracts covering different aspects of early chemotherapy in the treatment of rectal cancer. Osama Rahma et al. presented the results of the pembrolizumab arm from NRG-GI002 platform. NRG-GI002 is a basket study utilising a neoadjuvant score for assessment of activity of novel compounds in TNT. Despite a favourable toxicity profile, pembrolizumab added to neoadjuvant chemoradiation has not improved the early outcomes of locally advanced rectal cancer patients [39].

In conclusion, the definition of “new standards of care” in locally advanced rectal cancer and anal cancer is ongoing: It is clear now that many treatment modalities may be subject to decision making by clinical factors as well as radiographic assessment, and a large armamentarium of (situation dependend) “best options” are available, also in consideration of the treatment goals. However, the role of newer decision factors—mostly from biologic factors—is still matter of investigation.

## Highlights in metastatic colorectal cancer

### EORTC metastatic colorectal cancer task force: Thibaud Kössler, Francesco Sclafani, Mark Peeters

**Fig. 4:**
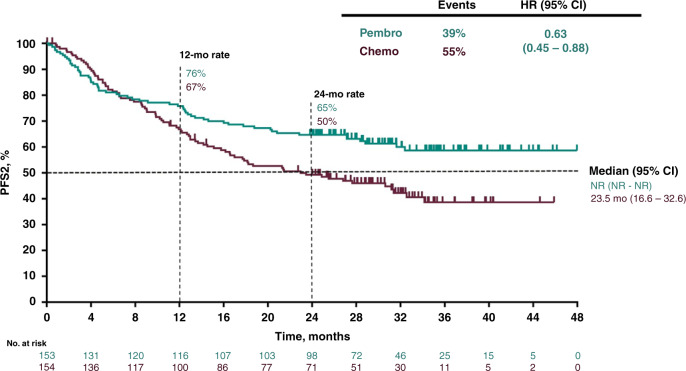
Time from randomisation to progression on next line of therapy (PFS2) from the KEYNOTE-177 trial: Pembrolizumab versus chemotherapy for microsatellite instability-high advanced colorectal cancer. Abstract 6 presented by Dr K-K Shiu at 2021 ASCO Gastrointestinal Cancers Symposium [41]. Reproduction with the permission of the author.

The KEYNOTE-177 is a randomised phase III trial comparing pembrolizumab (anti-PD-1) to chemotherapy in MSI-High/dMMR metastatic CRC (mCRC) in first line. Pembrolizumab showed improvement in progression-free survival (PFS, co-primary outcome) (median, 16.5 vs. 8.2 months; HR 0.60; 95% CI, 0.45–0.80, *P* = 0.0002, overall RR: complete and partial, 43.8% vs. 33.1% and ≥ grade 3 treatment-related adverse events (TRAEs) (22% vs. 66%) [40]. At ASCO-GI 2021, the PFS2 (PFS on next line of therapy or death from any cause) was presented and also favoured pembrolizumab (NR vs. 23.5 months; HR 0.63, 95% CI, 0.45–0.88), Fig. 4. Quality of life was also significantly improved in the immunotherapy arm using the EORTC QLQ -C30 scale [41]. OS, the second co-primary outcome, are expected in 2021. These results confirm pembrolizumab as standard of care, for first-line treatment in MSI-High/dMMR mCRC.

he GARNET trial is testing dostarlimab (anti-PD-1) in a multi-cohort trial. This year the non-endometrial dMMR/MSI-H pan tumour cohort was presented. Primary objectives were ORR and duration of response (DOR). Amongst 99 patients recruited, ORR was 38.7% (95% CI, 29.4–48.6) with complete response in 7.5%. The median DOR was not reached and grade ≥ 3 TRAEs were 8%. Within the 69 (65%) mCRC enrolled, ORR was 36.2%, matching the results of other anti-PD-1 antibodies (nivolumab and pembrolizumab) in pre-treated dMMR/MSI-H mCRC [42–44]. These results confirm the activity of anti-PD-1 antibodies in pre-treated MSI-High/dMMR mCRC.

Preclinical data have shown that anti-EGFR therapy (e.g. panitumumab or cetuximab) causes tumour-specific adaptive immune response and immunogenic apoptosis, with functional adaptive immunity required to mediate efficacy [45]. Anti-EGFR antibody therapy is associated with increased expression of CTLA-4 and PD-L1 [46]. The LCCC1632 trial, hypothesised that addition of ipilimumab (anti-CTLA-4) and nivolumab (anti-PD-1) to panitumumab increases response rate (at 12 weeks) in pre-treated patients with *KRAS*/*NRAS*/*BRAF* WT MSS mCRC. The trial enrolled 56 patients; out of the 49 evaluable subjects, the 12-week RR was 35% (95% CI, 21–48) with no complete response, median PFS was 5.7 months (95% CI, 5.5–7.9) and median OS was 27 months (95% CI, 14.5-NR) [47]. These results show no advantage over anti-EGFR treatment alone [48].

The TASCO1 study is a phase II randomised open-label noncomparative study assessing safety and efficacy (PFS, primary outcome) of first-line treatment with trifluridine/tipiracil plus bevacizumab (TT-B) and capecitabine plus bevacizumab (C-B) in untreated patients with mCRC not candidates for standard oxaliplatin- or irinotecan-based chemotherapy regimens. Median PFS was 9.2 (95% CI, 7.6–11.6) and 7.8 (95% CI, 5.5–10.1) months in the TT-B and C-B groups, respectively [49]. At ASCO-GI 2021, authors presented the median OS which was 22.31 months in TT-B and 17.67 months in C-B with HR 0.78 (95% CI, 0.5–1.10). Survival probability at 18 months in TT-B was 62% (95% CI, 50–72%), and 47% (95% CI, 35–57%) in C-B [50]. TT-B treatment shows clinical activity in untreated patients with unresectable mCRC ineligible for intensive therapy.

In conclusion, MSI-H/dMMR mCRC patients should receive immune checkpoint inhibitors as first (preferably) or later lines of treatment. One of the burning questions is how to select those who will respond to single-agent immune checkpoint inhibition and those who would benefit from double immunotherapy (the combination of anti-PD-1 + anti-CTLA-4 likely improving outcomes as suggested by the single-arm CheckMate-142 study) or standard chemotherapy. Furthermore, future research efforts should be largely directed to the investigation of alternative treatment strategies that could extend the benefit of immunotherapy to the by far larger group of inherently immune-resistant MSI-H/dMMR mCRC patients.

## Highlights in early phase trials

### EORTC early phase trials task force: Radka Obermannova and Maria Alsina

Several new initiated early phase studies and phase 3 combinations of target therapies were presented at the congress. The following account provides an overview of selected trials in progress investigating new molecules, especially target treatment and immunotherapy combinations.

In HER2 positive disease, phase II/III study MOUNTAINEER-02 investigates tucatinib, trastuzumab, ramucirumab and paclitaxel in previously treated HER2-positive gastric or gastroesophageal junction adenocarcinoma (GEC). Tucatinib is a highly selective HER2-directed TKI. Since phase 2 will establish a recommended dose of combination, phase 3 aims to compare the efficacy and safety of tucatinib plus trastuzumab (Arm 3 A; 235 patients) vs. placebo (Arm 3B;235 patients), both in combination with ramucirumab and paclitaxel, and also evaluate the activity of tucatinib, ramucirumab, and paclitaxel (Arm 3 C; 30 patients). The dual primary phase 3 endpoints are OS and PFS [51].

DESTINY-Gastric03 is a phase Ib/II, dose-escalation, and dose-expansion study that evaluates trastuzumab deruxtecan (T-DXd, DS-8201) monotherapy and combinations with chemotherapy or ±checkpoint inhibitor in patients with HER2-overexpressing gastric cancer. In part 2, the stratification according to HER2 status will be provided (NCT04379596). Finally, an ongoing study with margetuximab combined with anti-PD-1 (retifanlimab) or anti-PD-1/LAG-3 (tebotelimab) ± chemotherapy in first-line therapy of advanced/metastatic HER2 + gastroesophageal junction (GEJ) or gastric cancer (GC) deserves mention [52].

Checkpoint inhibitors showed modest activity in unselected population in gastric cancer. NCT04164979 is the prospective, phase 2 trial investigating if cabozantinib contributes to overcoming primary or secondary resistance to PD-1 blockade in GEC. Patients previously treated with fluoropyrimidine/platinum and a prior checkpoint inhibitor if tumour PD-L1 CPS ≥ 10% are assigned to cabozantinib plus pembrolizumab. The primary objective is determining the feasibility of the combination and estimating its efficacy [53].

Another phase II/III study focuses on the treatment selected based on new predictors. Cohort 3 of the Phase II ILUSTRO study evaluates zolbetuximab, a chimeric IgG1 monoclonal antibody, against CLDN18.2 in combination with pembrolizumab in claudin 18.2 positive locally advanced or metastatic gastric or gastroesophageal junction adenocarcinoma with the hypothesis that this combination might augment ADCC and antitumour immune response in CLDN18.2 overexpressing cancers. The primary endpoint is the objective response rate (NCT03505320) [54].

NCT03207347 is a phase II, multicenter study aiming to exploit the concept of synthetic lethality using the PARP inhibitor niraparib in patients with metastatic relapsed refractory solid tumours. Cohort A includes tumours harboring suspected BAP1 mutations (in terms of histology, including cholangiocarcinoma, uveal melanoma, mesothelioma or clear cell renal cell carcinoma) with tissue available for BAP1 mutational assessment via NGS or Cohort B (histology-agnostic): tumours with known DNA damage response (DDR) mutations confirmed by CLIA-approved NGS [55].

NCT04515394 is a phase II study in patients with advanced left-sided *RAS*/*BRAF* wild-type colorectal cancer. This study aims to explore tepotinib plus cetuximab in patients with acquired resistance to anti-EGFR antibody therapy due to MET amplification. Tepotinib is an oral, highly selective, potent MET tyrosine kinase inhibitor. The primary endpoint is an investigator-assessed objective response [56].

And finally, the last selected study is SWOG S2001, a randomised phase II trial exploring maintenance olaparib monotherapy *versus* olaparib plus pembrolizumab in metastatic pancreatic cancer patients with germline BRCA 1 or BRCA 2 mutations who did not progress on first-line platinum-based chemotherapy. The primary endpoint is to reach mPFS of 11.7 months in the experimental arm (NCT04548752) [57].

In conclusion, based on known predictors, in HER2 positive metastatic GC, results from trials of trastuzumab deruxtecan, and similarly, margetuximab, in combination with checkpoint inhibitors should evaluated in the light of results from ongoing phase III KEYNOTE-811 trial (NCT03615326) that is focusing on trastuzumab and pembrolizumab combination, and then phase II INTEGA trial, exploring the combination of nivolumab and trastuzumab or chemotherapy plus ipilimumab (AIO STO 0217). Zolbetuximab plus checkpoint inhibitor in claudin 18.2 population will show if augments antitumour immune response and ADCC. And finally, tepotinib in combination with cetuximab could be a promising new target therapy in RAS wt CRC with acquired resistence to anti-EGFR therapy.

## Highlights in adolescent and young adults

### EORTC adolescent and young adults task force: Irit Ben-Aharon

Early-onset colorectal cancer (EOCRC) represents a major clinical and health-policy challenge due to its rising incidence in the Western world in the past decade. A study performed by the Colorectal Cancer Alliance addressed the results of an online survey of EOCRC patients and survivors (<50 y) in the US, which was designated to capture their self-reported experiences using several quality-of-life validated tools [58]. Among 1089 participants, the majority were female and Caucasian. Most respondents (62%) waited 3–12 months before seeking medical advice, and most indicated they were initially misdiagnosed or referred to multiple doctors prior to cancer diagnosis. African-Americans were four times more likely to report poor health compared with Caucasian respondents. Asian Pacific and Hispanic-Latino reported favourable prior health status. More than 77% of the respondents were diagnosed at stage 3 or 4 disease. These results imply multiple challenges in the primary care of young patients diagnosed with CRC manifested by a delayed path to diagnosis and subsequently advanced stage at diagnosis. Another study evaluated racial disparities among EOCRC compared with older-onset [59]. Colorectal cancer patient data were retrieved from the National Cancer Database (NCD) and analyzed upon age and other sociodemographic parameters. The study population comprised 1,061,204 patients, 10.2% were EOCRC. Compared to older patients, EOCRC patients were more likely to be African-American or Hispanic and to be diagnosed with metastatic disease. Young patients had improved survival over older patients (median OS 157.4 months compared with 64.2 months).

Upper gastrointestinal cancers such as esophageal and gastric cancer are on the rise, while there is a paucity of data regarding the disease of early-onset esophagogastric cancer (EOEGC). A retrospective study evaluated clinical features and molecular landscape of EOEGC compared with average-onset and revealed data on 151 EOEGC (<49 y) and 587 average-onset patients [60]. Time to diagnosis was longer in EOEGC, though stage did not differ. Signet cell histology was more common in EOEGC. A trend toward higher MSI-High status in average-onset was demonstrated. Another study addressed socioeconomic and pathologic characteristics of EOEGC in the NCD and revealed that EOEGC (defined < 60 y) tend to be African-American or Hispanic and to have an advanced disease at presentation [61]. An optimistic note with regard to early-onset pancreatic cancer (EOPC) derives from a study which appraised clinical characteristics of EOPC (<60 y) retrieved from the NCD and compared to average-onset. EOPC patients had higher median OS, though they were more likely to be diagnosed with metastatic disease [62].

In conclusion, as the incidence of early-onset GI cancers is rising, elucidating epidemiological and biological perspectives is key for early detection and treatment tailoring. Studies presented herein which addressed self-reported experiences of EOCRC patients revealed a delayed course to diagnosis and subsequently late stage, as well as racial disparities that was also demonstrated in early-onset gastric cancer. An optimistic view reflected by current data indicating that survival of early-onset GI cancer patients is not inferior to average-onset patients.

## Conclusion

### EORTC Gastrointestinal Tract Cancer Group: Markus Moehler (Group Chairman) and Elisa Fontana (Group Secretary)

Significant efforts to find effective treatment options aimed at improving survival outcomes in gastrointestinal cancer continue, despite the COVID-19 pandemic. Drug development and clinical trial machinery should not stop; similarly, reporting clinical trial results and sharing details of trials in progress with the research community is crucial. Switching to virtual meetings may possibly have had an impact on conference attendance, the exchange of ideas, and interactions between academic researchers and pharmacompanies. Attendance at key events should be encouraged and facilitated; with this manuscript we want to support the divulging of new scientific findings presented in a key conference for physicians treating gastrointestinal cancers.

The importance of targeting key molecular events like HER2 overexpression or FGFR gene alteration is once again highlighted in this conference. Adding targeted agents to the backbone of chemotherapy regimens in early lines seems feasible although possibly challenged by biomarker testing; turnaround time and tissue availability need to be taken into account as soon as possible for timely clinical trial inclusion. Chemo-free approaches are more frequently explored; single-agent activity of FGFR inhibitors is well known in upper GI and biliary track cancers. The development of new-generation anti-HER2 agents like margetuximab and zanidatamab, with proven single-agent activity, may expand chemo-free opportunities also in HER2 positive cancers.

Immune checkpoint inhibitor (CPI) activity continues to be explored in combination with chemotherapy and targeted agents or in chemo-free regimens, with interesting results in upper GI and hepatobiliary cancers. In colorectal cancer, the significant impact of CPIs in microsatellite instable cancers was once again confirmed in both first-line and chemorefractory setting; conversely, another attempt to switch cold microsatellite stable CRC into hot tumours was disappointing.

In line with global efforts, within our GI Task Forces several ongoing trials are exploring HER2-targeted and CPIs approaches. The INNOVATION trial is exploring whether adding trastuzumab or trastuzumab and pertuzumab to chemotherapy increases the pathological response rate in HER2 positive gastric or gastroesophageal adenocarcinomas (NCT02205047). The adjuvant VESTIGE trial is investigating nivolumab plus ipilimumab versus standard post-operative chemotherapy in gastroesophageal adenocarcinoma patients with high risk of disease recurrence (NCT03443856). The ABC-09 trial is an open-label single-arm trial of pembrolizumab plus standard first-line chemotherapy in biliary track cancers (NCT03260712). The ILOC trial is a phase II trial of durvalumab plus local tumour ablation in patients with colorectal liver metastases (NCT03101475).

## Data Availability

All data were presented during the 2021 Virtual Gastrointestinal Cancers Symposium held between 15 January and 17 January 2021. All data are available from https://meetinglibrary.asco.org/browse-meetings/2021%20Gastrointestinal%20Cancers%20Symposium.
